# A prospective, randomized, controlled study of ω-3 fish oil fat emulsion-based parenteral nutrition for patients following surgical resection of gastric tumors

**DOI:** 10.1186/1475-2891-13-25

**Published:** 2014-03-24

**Authors:** Ziran Wei, Weimin Wang, Ji Chen, Dejun Yang, Ronglin Yan, Qingping Cai

**Affiliations:** 1 Department of General Surgery, Shanghai Changzheng Hospital, Second Military Medical University, Shanghai, China

**Keywords:** ω-3 fish oil, Immune nutrition, Eicosapentaenoic acid (EPA), Docosahexaenoic acid (DHA), Gastric tumor, Parenteral nutrition

## Abstract

**Background:**

Nutrients such as ω-3 fatty acids including fish oil components eicosapentaenoic acid (EPA) and docosahexaenoic acid (DHA) suppress the growth and promote apoptosis of tumor cells, improve immune function and reduce the effects of systemic inflammatory response syndrome. We sought to investigate the effect of ω-3 fish oil fat emulsion-based parenteral nutrition (PN) on nutritional state, immune function, inflammatory reaction, expression of tumor factors and complication incidence in patients after surgical resection of gastric cancer.

**Methods:**

Forty-eight patients after surgical operation of gastric tumor in hospital were randomly divided into the control group and intervention group. Patients in both groups were treated with iso-nitrogen and iso-caloric parenteral nutrition support. In addition, the intervention group received ω-3 fish oil fat emulsion and the control group received soybean oil. The indicators of nutrition, immune function and inflammation in the two groups were detected on the day before the operation and postoperative day 6. The rate of complication was compared between the two groups.

**Results:**

There was no significant difference in nutritional state, liver function and renal function between the two groups (P > 0.05). However, the levels of inflammatory markers were significantly decreased (P < 0.01), and the rate of complication was also decreased in the intervention group as compared with the control group.

**Conclusions:**

ω-3 fish oil fat emulsion-based parenteral nutrition alleviates the inflammatory reaction and reduces the rate of inflammatory complications.

## Background

Omega-3 polyunsaturated fatty acids including fish oil components eicosapentaenoic acid (EPA) and docosahexaenoic acid (DHA) are essential for humans as they cannot be synthesized by the human body. There have been an increasing number of studies on immune nutrition and cancer therapy over the recent years, and preliminary data suggest that EPA and DHA suppress the growth and promote apoptosis of tumor cells, as well as improve immune function and reduce the effects of systemic inflammatory response syndrome [1-5]. A recent survey revealed that the content of polyunsaturated fatty acids is likely inadequate in the regular diet of Chinese [6]. A randomized controlled study evaluated the effect of omega-3 fatty acid-supplemented total parenteral nutrition (TPN) on the clinical outcomes of patients with hepatitis B virus (HBV)-associated hepatocellular carcinoma (HCC) showed that postoperative administration of omega-3 fatty acid reduced infection and improved postoperative hepatic function recovery [7]. Short-term pre-operative infusion of fish oil alone has been shown to improve the postoperative immune response of gastrointestinal cancer patients but exhibited no apparent effect on postoperative infections or length of intensive care unit (ICU) and hospital stay [8]. A recent meta analysis evaluated n-3 PUFA-enriched parenteral nutrition (PN) regimens in elective surgical and ICU patients and showed that these regimens are safe and effective in reducing the infection rate and hospital/ICU stay in surgical and ICU patients [9].

The above findings prompted us to speculate that f ω-3 fish oil fat emulsion-supplemented PN could be beneficial to the overall functional recovery of gastric tumor patients. Here, we designed a prospective, randomized, single-blinded control clinical trial to investigate the effect of ω-3 fish oil fat emulsion-supplemented PN on nutritional state, immune function, inflammatory reaction, expression of tumor factors and complication incidence in patients after surgery of gastric tumors.

## Methods

### Patient selection

This prospective, randomized, controlled, single center study was carried out at the authors’ affiliated hospital between May 2007 and March 2008. The study protocol was approved by the university and hospital ethics committees and written informed consent was obtained from all study subjects or their legal surrogates. The study was carried out in accordance with the Helsinki Declaration. Complete history was taken and physical examinations were carried out. General assessment of nutritional status included measurements of body height, body weight and body mass index (BMI) (kg/m^2^). Exclusion criteria for the selection of patients included: (1) age < 18 or > 75 years; (2) BMI < 16 or > 30 kg/m^2^; (3) hepatic insufficiency (Child–Pugh grade B or above); (4) abnormal renal function (serum creatinine > 3 mg/dL); (5) ongoing infections and fever in the preceding month; (6) major gastrointestinal disease such as Crohn’s disease; (7) autoimmune disorders, steroid treatment, and medication which could modulate metabolism or body weight; (8) pregnancy or breast-feeding; (9) received total parenteral support 2 months before operation; (10) severely malnourished (weight loss of 10% of body weight during the previous 6 months).

### Nutrition

Postoperatively, all patients received TPN for at least 6 consecutive days through an indwelling central venous catheter. These patients were randomly divided into the intervention (26 patients) and control (26 patients) group. Both groups were given parenteral nutrition consisting of 104–125 kJ/kg/d of calories for energy with glucose and fat emulsion as the main sources of energy. Fat emulsion intralipid (20% Intralipid, Sino-Swed Pharmaceutical Corp. Ltd.) made up 35–50% of the total calories provided and 0.15–0.20 g/(kg d) of nitrogen was provided using 8.5% Novamin^®^ (Sino-Swed Pharmaceutical Corp. Ltd.). Glucose and exogenous insulin were provided at a ratio of 6:1, along with vitamins, water, electrolytes and trace elements via a peripheral vein or central venous infusion for 10 to 12 hours. Finger blood sugar was monitored every 4–6 hours, and if blood sugar was high, insulin was given to control glucose level. Patients in both groups received about 2800 mL liquid per day and without any intake per oral. In all patients, prophylactic antibiotic treatment was given for 3 to 4 days postoperatively (Cefuroxime, 2 g b.i.d. intravenously (i.v.) and metronidazole 500 mg b.i.d. i.v.). In the control group (Group I), the fat emulsion used was omega-6 lipid (20% Intralipid, Sino-Swed Pharmaceutical Corp. Ltd.). In the intervention group (Group II), the omega-6 lipid content of TPN was partially replaced by omega-3 PUFA (10% Omegaven, Sino-Swed Pharmaceutical Corp. Ltd.) up to 0.2 g/kg body weight daily; therefore, the omega-3/omega-6 ratio was 1:4. Calculated by body mass, nutrition in both groups was isonitrogenous and isocaloric.

### Blood sample assays

In the morning before operation and on postoperative day 6, blood sample assays were collected from all the patients. Routine blood test and biochemistry analysis including total protein (TP), albumin (ALB), pre-albumin (PA), retinol binding protein (RBP), transferrin (TF), serum total cholesterol (TC), total bilirubin (TBil), alanine aminotransferase (ALT), aspartate aminotransferase (AST) and creatinine (Cr) were immediately performed at the Department of Clinical Chemistry, Shanghai Changzheng Hospital according to standard laboratory procedures.

Percentage of CD3+, CD4+, and CD8+ lymphocytes were analyzed by flow cytometry at the Department of Clinical Chemistry, Shanghai Changzheng Hospital according to standard laboratory procedures.

Serum was also separated from 3 mL blood from each patient at 4°C and centrifuged at 3000 rpm for 5 minutes. Serum from each patient was stored at -20°C and used to run an ELISA assay for quantification of serum IL-1β, IL-6, and TNF-α using the Model 550 Microplate Reader (American Bio-rad) in accordance with standard operating instructions.

### Patient monitoring

Postoperative complications including respiratory tract infections (if chest radiographic examination showed new or progressive infiltrates and temperature increases above 38.5°C), urinary tract infection (if urine culture showed at least 10^5^ colonies of a pathogen), abdominal abscess (if patient reported abdominal pain and high white blood cell count, and ultrasound examination detected an anechoic area in the abdomen), and wound complications, such as fistula and/or wound infection were assessed by the same investigator surgeon and recorded accordingly.

### Statistical analysis

Mean ± SD of each value was obtained and the difference between the intervention and control groups for each value tested for statistical significance using the software SPSS (version 13.0). Analysis of variance or Student’s *t*-test or chi-square test was used in statistical analyses. P < 0.05 was considered statistically significant.

## Results

### Patient demographic and baseline characteristics

Fifty-two patients with gastric tumor were enrolled including 26 males and 26 females aged between 29 and 75 years (median, 53.5 years). Six patients were excluded from the control group because of incomplete data and as a result there were 20 patients in the control group. All patients in the study did not suffer any severe complications or death. During hospitalization, the vital signs of both groups were stable. No adverse effects associated with parenteral nutritional support were observed such as parenteral nutrition-associated liver disease (PNALD), infection, phlebitis, and glucose intolerance. Patient demographic and baseline characteristics are shown in Table 1.

**Table 1:** Patient demographics and baseline characteristics

**Variables**	**The control group**	**The intervention group**	**P**
**Number**	20	26	
**Sex ratio (M:F)**	11:9	15:11	>0.05
Age (years)*	59(36–74)	50.5(29–75)	>0.05
**Height (cm)**	166.5(150–185)	168(155–183)	>0.05
**Weight (kg)**	65(45–89)	62(42–88)	>0.05
**Body mass index (kg/m**^**2**^)*	22.5(17.8–29.7)	22.2(15.7–28.1)	>0.05
**TNM stage**			
**I**	11	13	>0.05
**II**	9	13	

*Values are median (range).

### Changes in the nutritional indices

There were no significant differences (P > 0.05) between the two patient groups in nutritional indicators such as TP (g/L), ALB (g/L), PA (mg/L), RBP (g/L), TF (g/L) and TC (mmol/L) before and after operation as shown in Table 2. However, TP, ALB, PA, TF and TC were significantly reduced after surgery in the intervention group, and ALB and TF were significantly decreased in the control group (indicated by * in Table 2).

**Table 2:** Comparison of pre- and postoperative nutritional indices of the study patients

**Indices**	**Preoperative**			**Postoperative**		
	**Study group**	**Control group**	**P**	**Study group**	**Control group**	**P**
TP	68.97 ± 6.38	68.35 ± 7.66	0.77	64.58 ± 6.14^*^	64.88 ± 6.47	0.87
ALB	41.52 ± 3.89	39.26 ± 4.52	0.08	37.17 ± 6.16^*^	36.07 ± 4.63^*^	0.49
PA	242.96 ± 66.25	218.03 ± 50.36	0.15	191.42 ± 55.31^*^	197.63 ± 41.65	0.67
RBP	0.034 ± 0.012	0.028 ± 0.010	0.07	0.049 ± 0.058	0.032 ± 0.011	0.15
TF	2.577 ± 0.63	2.447 ± 0.48	0.44	1.934 ± 0.45^*^	1.93 ± 0.42^*^	0.97
TC	3.84 ± 0.61	4.22 ± 1.06	0.16	3.29 ± 0.72^*^	3.85 ± 1.64	0.17

TP, total protein; ALB, albumin; PA, pre-albumin; RBP, retinol binding protein; TF, transferrin; TC, total cholesterol.

*Indicates significant difference vs. the preoperative value in the same group.

### Changes of immunologic parameters

There were no significant differences (P > 0.05) between the two groups in immunologic indicators such as total T lymphocyte cells (TLC, ×10^9^/L), CD3(%), CD4(%), CD8(%) and CD4/CD8 positive lymphocytes before surgery. Postoperatively, all these parameters remained comparable (Table 3). Comparing the preoperative and postoperative data, these parameters were not statistically different in either the control or intervention group.

**Table 3:** Comparison of the pre- and postoperative immune indices of the study patients

**Indices**	**Preoperative**			**Postoperative**		
	**The intervention group**	**The control group**	**P**	**The intervention group**	**The control group**	**P**
TLC	1.31 ± 0.42	1.28 ± 0.41	0.701	1.12 ± 0.37	1.08 ± 0.36	0.438
CD3	67.14 ± 8.58	65.74 ± 9.20	0.599	64.71 ± 10.51	66.07 ± 7.64	0.615
CD4	42.48 ± 8.91	38.92 ± 9.80	0.211	44.23 ± 9.46	41.74 ± 8.67	0.358
CD8	22.69 ± 8.85	23.92 ± 7.57	0.61	19.85 ± 7.36	21.47 ± 5.94	0.414
CD4/CD8	2.34 ± 1.19	1.90 ± 1.06	0.212	2.61 ± 1.33	2.16 ± 1.00	0.198

TLC, total T lymphocyte cells.

### Inflammatory response

There were no statistical differences (P > 0.05) between the two groups of patients in the five inflammatory indicators – WBC (×10^9^/L), CRP (mg/L), IL-1β (pg/mL), IL-6 (pg/mL) and TNF-α (pg/mL) before surgery. The differences in WBC, IL-1β, IL-6 and TNF-α between the 2 groups post surgery were statistically significant (P < 0.05), as shown in Table 4. Comparing the preoperative and postoperative data, WBC, IL-6 and TNF-α of the intervention group did not increase, whereas those of the control group had a significant increase (indicated by * in Table 4). CRP and IL-1β of both groups increased significantly after surgery.

**Table 4:** Comparison of the pre- and postoperative inflammatory indices of the study patients

**Indices**	**Preoperative**			**Postoperative**		
	**Study group**	**Control group**	**P**	**Study group**	**Control group**	**P**
WBC	5.26 ± 3.09	4.76 ± 1.37	0.331	7.42 ± 2.79	5.95 ± 1.46^*^	0.025^*^
CRP	3.98 ± 2.17	5.64 ± 9.83	0.269	30.90 ± 31.17^*^	33.62 ± 7.36^*^	0.733
IL-1β	201.51 ± 16.06	196.38 ± 21.90	0.422	302.32 ± 37.61^*^	332.61 ± 35.34^*^	0.011^*^
IL-6	15.53 ± 61.57	16.56 ± 62.04	0.950	14.35 ± 45.95	48.25 ± 67.49^*^	0.007^*^
TNF-α	15.31 ± 52.61	19.77 ± 52.38	0.889	12.05 ± 60.31	53.56 ± 75.23^*^	0.005^*^

CRP. C-reactive protein; WBC, white blood cells.

*Indicates significant difference vs. the preoperative value in the same group.

### Expression of tumor-related factors

There was no statistical difference between the two groups in VEGF (pg/mL) and IGF-1 (ng/mL) before and after surgery (P > 0.05), as shown in Table 5. Comparing the pre-surgery and post-surgery data, VEGF and IGF-1 of both groups showed a trend of decrease, but no statistically significant difference was observed.

**Table 5:** Comparison of the preoperative and postoperative tumor factors of the study patients

**Indices**	**Preoperative**			**Postoperative**		
	**The intervention group**	**The control group**	**P**	**The intervention group**	**The control group**	**P**
VEGF	346.21 ± 366.55	271.08 ± 276.68	0.389	200.35 ± 183.61	177.30 ± 167.83	0.622
IGF-1	403.31 ± 28.50	393.81 ± 34.53	0.316	369.13 ± 53.23	341.87 ± 75.27	0.119

### Postoperative infectious complications

There were three cases of incisional wound infection, one case of abdominal infection, one case of urinary infection and one case of pulmonary infection in the control group; and there was one case of incisional wound infection and one case of pulmonary infection in the intervention group. Although the comparison of each kind of complication did not shown significant difference between the two groups, the combined incidences of complications in the control group was significantly higher than that of the intervention group, as shown in Table 6.

**Table 6:** Comparison of postoperative infectious complications of the study patients

	**The control group**	**The intervention group**	**Fisher’s exact test (two-tailed)**
Incisional wound infection	3/20	1/26	P = 0.303
Abdominal infection	1/20	0/26	P = 0.435
Urinary infection	1/20	0/26	P = 0.435
Pulmonary infection	1/20	0/26	P = 0.435
**Total complications**	**6/20**	**1/26**	**P = 0.033**

## Discussion

Cancer is a wasting disease and cancer patients are at a high risk of malnutrition. Due to the possibility that parenteral nutrition may stimulate tumor growth and proliferation, parenteral nutritional support for tumor patients is controversial [10]. However, it is clear that nutritional support is important for patients undergoing surgery or receiving chemotherapy, radiation therapy and other cancer treatments, which also helps maintain the body’s immunity. Appropriate perioperative nutritional support is important in improving the nutritional status of the body, maintaining the structure and function of the organs, reducing the incidence of surgical mortality and postoperative complications, and improving the patient’s quality of life [11]. It also provides a good platform for the body to withstand the next round of treatment [11]. Our clinical trial suggests that a standard calorie parenteral nutrition program such as the one used in the current trial can effectively maintain the nutritional status of patients post surgery.

ω-3 fish oil is made up of the fatty acids EPA and DHA, which the body itself can only synthesize a very small amount. EPA and DHA can increase the amount of phospholipid omega-3 in cell membranes, while reducing inflammatory eicosane generation by increasing the amount of non-inflammatory eicosane competing in the arachidonic acid synthetic pathway. The release of antibodies and the phagocytic activity of macrophages are subsequently enhanced, thereby inhibiting the release of pro-inflammatory cytokines IL-1, IL-6 and TNF-α. As shown in our study in Table 4, IL-6 and TNF-α were only increased in the control group postoperatively, but not in the intervention group. The use of omega-3 fat emulsion can also increase the release of anti- inflammatory cytokines IL-10, IL- 13, and TGB-β, thus blocking the body’s excessive inflammatory response and reducing the incidence of Systemic Inflammatory Response Syndrome (SIRS) and Multiple Organ Dysfunction Syndrome (MODS). The mortality of patients was therefore decreased and the prognosis of patients improved after major abdominal surgery, and in patients with peritonitis and abdominal sepsis and other conditions [12]. In our study, we found a significantly higher chance of postoperative infections in the control group (Table 6), which is consistent with the higher postoperative WBC levels in the control group. Together, our data suggests that ω-3 fish oil fat emulsion supplementation can reduce the body’s pro-inflammatory reaction after surgery, contributing to lower incidences of postoperative infection and aiding in the early recovery of patients.

Current research on the effects of ω-3 fatty acids on immune function revolves around their effects on cytokines, adhesion molecules, differentiation antigen receptor expression, free radicals, peroxides, antibody production, lymphocyte proliferation, antigen-presenting cell function and natural killer cell cytotoxicity [13]. The role of ω-3 fatty acids in regulating the levels of these immune factors in the body can therefore lead to enhanced immune function [13]. Studies also suggest that ω-3 fatty acids may alter the lipid environment of membrane micro-lipids, shifting the receptor protein to a non-functional region, thereby generating immune-modulatory effects [14]. ω-3 fatty acids can also play its role in the immune system by modulating dendritic cells (DCs) [15].

Alanine aminotransferase and aspartate aminotransferase were increased in both groups 6 days post surgery, but the increase was to a lesser extent in the intervention group, which stayed within the normal range. This indicates that ω-3 fish oil fat emulsion may decrease the amount of hepatic damage resulting from gastric cancer surgery. This may be related to the role of ω-3 fatty acids in increasing liver blood perfusion and reducing intestinal bacterial translocation [16]; as well as their ability to reduce the release of prostaglandin E2 (PGE2), leukotriene B4 (LTB4) and platelet activating factor, resulting in the inhibition of L-1β, IL-2 and TNF-α mRNA expression, thereby blocking the excessive inflammatory response to protect the function of vital organs [17].

In conclusion, ω-3 fish oil fat emulsion-based parenteral nutrition promotes the recovery of the immune function, alleviates inflammation and reduces the rate of complications in patients with surgically resected gastric tumors. However, our findings await confirmation from future prospective controlled studies on a larger patient population.

## Abbreviations

ALB: Albumin; ALT: Alanine aminotransferase; AST: Aspartate aminotransferase; Cr: Creatinine; CRP: C-Reactive protein; DHA: Docosahexaenoic acid; EPA: Eicosapentaenoic acid; IL-1: Interleukin-1; LTB4: Leukotriene B4; MODS: Multiple organ dysfunction ssyndrome; PUFA: Polyunsaturated fatty acid; PA: Pre-albumin; PGE2: Prostaglandin E2; PN: Parenteral nutrition; RBP: Retinol binding protein; SIRS: Systemic inflammatory response syndrome; TB4: Leukotriene B4; TBil: Total bilirubin; TC: Serum total cholesterol; TF: Transferrin; TNF-α: Tumor necrosis factor-alpha; TP: Total protein L; TPN: Triphospho-pyridine-nucleotide.

## Competing interests

The authors declare that they have no competing interests.

## Authors’ contributions

All of the authors have made substantial contributions to the study ZW: (1) the conception and design of the study, acquisition of data, analysis and interpretation of data, (2) drafting the article. (3) final approval of the version to be submitted. WW: (1) the conception and design of the study, and interpretation of data, (2)revising the manuscript critically for important intellectual content, (3) final approval of the version to be submitted. JC: (1) interpretation of data, (2) revising the manuscript critically for important intellectual content, (3) final approval of the version to be submitted. DY: (1) the conception and design of the study, acquisition of data, analysis and interpretation of data, (2) revising the manuscript critically for important intellectual content, (3) final approval of the version to be submitted. RY: (1) interpretation of data, (2) revising the manuscript critically for important intellectual content, (3) final approval of the version to be submitted. QC: (1) the conception and design of the study, acquisition of data, analysis and interpretation of data, (2) revising the manuscript critically for important intellectual content, (3) final approval of the version to be submitted. All authors read and approved the final manuscript.
